# Dynamic blebbing: A bottleneck to human embryonic stem cell culture that can be overcome by Laminin-Integrin signaling

**DOI:** 10.1016/j.scr.2018.10.022

**Published:** 2018-11-02

**Authors:** Nikki Jo-Hao Weng, Cindy Cheung, Prue Talbot

**Affiliations:** a Department of Molecular, Cell and Systems Biology, University of California, Riverside, CA 92521, United States; b Cell Molecular and Developmental Biology Graduate Program, University of California, Riverside, CA 92521, United States

**Keywords:** Pluripotent stem cells, Blebbing, Laminin, Cell culture

## Abstract

This study characterizes dynamic and apoptotic blebbing in human embryonic stem cells (hESC), identifies dynamic blebbing as a bottleneck to successful cell attachment during passaging, and demonstrates that dynamic blebbing can be rapidly stopped by plating cells on recombinant human laminin. In freshly plated hESC, dynamic and apoptotic blebbing differed in time of occurrence, bleb retraction rate, mitochondrial membrane potential, and caspase 3&7 activation. While dynamic blebbing can be controlled with drugs that inhibit myosin II, these methods have off-target effects and are not suitable for clinical applications. Recombinant human laminin-521 or addition of laminin-111 to Matrigel provided a safe method to drastically decrease dynamic blebbing and improve cell attachment with proteins normally found in the inner cell mass. Inhibition of focal adhesion kinase, which is activated by binding of integrins to laminin, prolonged dynamic blebbing and inhibited attachment. These data show that hESC bind rapidly to laminins through an integrin, which activates focal adhesion kinase that in turn downregulates dynamic blebbing. Laminins enabled hESC to rapidly attach during passaging, improved plating efficiency, enabled passaging of single pluripotent stem cells, and avoided use of inhibitors that have non-specific off-target effects. These data provide a strategy for improving hESC culture using biologically safe recombinant human proteins.

## 1. Introduction

Human embryonic stem cells (hESC) were derived in 1998 (Thomson et al., 1998), 16 years after their first mouse counterparts were reported (Martin, 1981; Evans and Kaufman, 1981). hESC are generally derived from spare blastocysts offered for research purposes by patients undergoing *in vitro* fertilization (Thomson et al., 1998). Originally, hESC were cultured on mouse embryonic fibroblasts. However, many groups have worked on developing new protocols that do not need non-human components for hESC culture (Xu et al., 2001; Ludwig et al., 2006a). Two major improvements in hESC culture were the replacement of feeder layers with Matrigel, a hESC-qualified matrix, and the introduction of better defined, feeder-free maintenance culture media, such as mTeSR (Ludwig et al., 2006a; Ludwig et al., 2006b; McElroy and Reijo Pera, 2008; Hughes et al., 2010). In spite of these improvements, hESC do not readily attach to substrates and cannot easily be plated as single cells.

Blebbing, which occurs during passaging, is the major bottleneck to attachment of hESC to substrates. Cell blebs can be either dynamic (non-apoptotic) or apoptotic. Apoptotic blebs occur on the surfaces of cells during death and have been reported in numerous studies (Coleman et al., 2011; Cocca et al., 2002; Barros et al., 2003). Dynamic blebs are membrane protrusions that appear and disappear from the surface of healthy cells (Charras and Paluch, 2008). Dynamic blebbing occurs in three phases referred to as nucleation, expansion, and retraction (Charras, 2008). During nucleation, blebs begin to form when small areas of the plasma membrane detach from the cortical actin or when a local rupture occurs in the cortical actin. Once a bleb is nucleated, hydrostatic pressure in the cytoplasm drives bleb expansion causing cytosol to flow into the developing bleb (Charras, 2008). During expansion, the plasma membrane detaches further from the cortex, increasing bleb size. As bleb expansion slows, a new actin cortex reforms under the bleb membrane, and myosin II is recruited to the bleb to power retraction. Dynamic blebbing is a normal process during cytokinesis, when blebs appear at the poles of dividing cells (Boss, 1955; Porter et al., 1973; Fishkind et al., 1991; Boucrot and Kirchhausen, 2007; Hickson et al., 2006; Charras et al., 2006), and in some cells, dynamic blebbing is the driving force that enables cell migration (Tokumitsu and Maramorosch, 1967). Therefore, dynamic blebbing appears to be an important physiological process in certain circumstances. Dynamic blebbing also plays a role in some diseases. For example, blebbing provides the motive force for invasion of tissue by *Entamoeba histyltica* and migration of breast cancer cells during metastasis (Khajah and Luqmani, 2015).

Like many other cell types, dissociated single hESC form a number of blebs on their surfaces during passaging (Ohgushi et al., 2010; Weng et al., 2015; Guan et al., 2013; Guan et al., 2015a; Guan et al., 2015b). Blebbing of hESC begins during passaging when colonies are dissociated into single cells or small colonies. hESC that are undergoing vigorous dynamic blebbing do not attach well to Matrigel-coated dishes. Because hESC that fail to attach eventually undergo apoptosis, blebbing of hESC is sometimes considered to be apoptotic (Ohgushi and Sasai, 2011). Understanding and controlling blebbing in hESC is important as it decreases plating efficiency and hinders bulk production of hESC that would be needed in stem cell clinics for therapeutic applications. It also precludes plating of single cells for applications that require cell quantification, such as toxicological studies or drug testing.

To circumvent the bottleneck created by blebbing, Rho-associated protein kinase (ROCK) inhibitor (Y27632) is often used in hESC culture medium to facilitate attachment (Ohgushi et al., 2010; Watanabe et al., 2007; Harb et al., 2008; Krawetz et al., 2009). ROCK inhibitor suppresses blebbing by inhibiting ROCK which in turn inhibits non-muscle myosin II. However, ROCK inhibitors may not be optimal for several reasons. hESC appear morphologically abnormal and stressed during ROCK inhibitor treatment (Weng et al., 2015). Inhibition of the ROCK pathway can affect numerous cell processes (Amin et al., 2013), not just blebbing, and off-target effects may occur when Y27632 is used in hESC culture. Inclusion of ROCK inhibitor in hESC culture medium also alters IC_50_ values in toxicological studies (Fujimura et al., 2011) and may encourage unwanted differentiation of pluripotent cells (Kim et al., 2015). These factors may not be acceptable for culturing hESC that are eventually used for therapeutic purposes or in quantitative applications, such as drug testing.

The goals of this study were to characterize and compare dynamic and apoptotic blebbing in freshly plated hESC and to identify a safe method to reduce dynamic blebbing during passaging, thereby leading to improved culture protocols that will be suitable for all hESC applications.

## 2. Materials and methods

### 2.1. Inhibitors and depolymerizers

Nocodazole and cytochalasin D were purchased from Sigma Aldrich (St Louis, MO), and latrunculin A and swinholide A were gifts from Dr. Leah Haimo. Blebbistatin and ROCK inhibitors (Y27632 and H1152) were from Tocris Bioscience (Minnesota, USA). FAK inhibitor 14 was purchased from Sigma Aldrich (St Louis, MO) and Integrin α6/CD49f antibody was purchased from R&D systems (Minneapolis, MN).

### 2.2. Cell culture

Experiments were done using H9 hESC purchased from WiCell (Madison, WI). Before setting up experiments, hESC were expanded by plating on Matrigel-coated 6-well plates. Cultures were maintained in mTeSR medium (Stem Cell Technologies, Inc. Vancouver, Canada) in 5% CO_2_ at 37 °C and 95% relative humidity as described in detail previously (Lin and Talbot, 2011; Behar et al., 2012a; Behar et al., 2012b). When colonies reached 70%–80% confluency (about 1 to 1.5 million cells), hESC were used in experiments. For single cell experiments, cells were detached with Accutase (eBioscience, San Diego, CA) for 3 min. A 1 ml pipette was used to rinse cells off the plate by pipetting the Accutase repeatedly. Once the cells detached from the plate, Accutase was neutralized using mTeSR medium. To separate colonies into single cells, they were passed through an 18-gauge syringe needle and 150,000 cells were plated in 35 mm high culture dishes for live cell imaging (Ibidi, Wisconsin, USA). Dynamically blebbing cells were studied immediately after plating on Matrigel (Corning, NY) or laminin-521 (Biolamina, Sweden) coated-dishes, while apoptotically blebbing cells were studied after 1.5 h of incubation on non-coated dishes, which prevent attachment.

Mouse embryonic fibroblasts (mEF) were derived from 12.5-day to 13.5-day pregnant mouse using the ATCC protocol and then frozen in liquid nitrogen (Lin and Talbot, 2011; Behar et al., 2012a; Behar et al., 2012b). mEF were expanded by plating on 0.1% gelatin (Sigma 128-K-0066, St Louis MO.) in T-25 flasks. Cultures were maintained in MEF Medium (450 ml DMEM, 50 ml FCS, 5 ml 1 × l-glutamine, 5 ml 1 × non-essential amino acids, and 5 ml 1 × sodium pyruvate) in 5% CO_2_ at 37 °C. When MEFs were 90% confluent, they were used in experiments. MEFs were detached from flasks with 0.25% trypsin for 1.5 min. MEF medium was used to inactivate the trypsin. Cells were centrifuged at 1,200 rpm for 3 min, resuspended in MEF medium, and cells were plated in Ibidi culture dish for live-cell imaging.

D3 mouse embryonic stem cells (mESC) were purchased from ATCC (#CRL-11632, Manassas, VA) and used as described previously (Lin et al., 2009). All experiments were done with passages 9–24. D3 mESC were plated on mitotically inactivated mEFs in stem cell medium containing 81.5% DMEM, 15% FBS, 0.98% l-glutamine, 0.98% sodium pyruvate, 0.98% non-essential amino acids, 0.5% penicillin/streptomycin, 0.00065% beta-mercaptoethanol and 0.00025% leukemia Inhibitory factor (LIF). The medium was changed daily. Cells were used for experiments at 70–75% confluency.

The prostate cancer cells were a gift from Dr. Manuela Martins-Green (UCR) and were provided for a one-time use in their standard culture medium (Wang et al., 2011).

### 2.3. Use of live cell-imaging to compare dynamic blebbing in four cell types

A Nikon BioStation IM, which combines an incubator, microscope, and cooled CCD camera, was used to collect time-lapse video data (Weng et al., 2015; Talbot et al., 2014; Lin et al., 2015). To create time-lapse videos, human prostate cancer cells, mEF, mESC, and hESC were plated on 35 mm culture dishes and incubated in a BioStation IM. Frames were captured every 3 min for 6 h from 10 to 12 different fields. To quantify blebbing and cell attachment during the first 100 min of incubation, cells in videos were classified as blebbing or attached based on their morphology. The percentage of blebbing and attached cells were counted every 20 min for 100 min. To determine the number of blebs/cell, cells were randomly picked from five videos in three different experiments. > 50 cells were analyzed in each group. The number of blebs produced by the cells was counted manually. In some experiments, the intensity of dynamic and apoptotic blebs was analyzed using Image J.

### 2.4. Verification that live cells were healthy and dying cells were undergoing apoptosis

MitoTracker Red CMXRos (ThermoFisher Scientific, Waltham, MA) and the Magic Red Caspase 3&7 Assay Kit (ImmunoChemistry, Bloomington, MN) were used to evaluate cell health and apoptosis. Mitotracker is a red-fluorescent dye that enters the mitochondria in live cells; its accumulation is dependent on the mitochondrial membrane potential. The Magic Red kit measures apoptosis by detecting active forms of caspase 3 and 7 in living cells. H9 hESC were loaded with 250 nM of Mitotracker or Magic Red during plating on a 35 mm dish in a BioStation IM. Phase contrast and fluorescent images were captured at 40× magnification every 10 min for 12 h from 10 different fields in each experiment. A total of 3 experiments was performed.

### 2.5. Comparison of the rate of bleb formation and retraction in dynamic and apoptotic cells

Single hESC were incubated on non-coated 35 mm dishes in a BioStation IM. Real time videos (30 fps) were collected from the BioStation monitor using a Canon 1080p HD Video Camcorder (Melville, NY). The rates of bleb formation and retraction were analyzed between 0 and 1 h and between 1.5 and 2.5 h for dynamic and apoptotic blebbing, respectively. Videos were analyzed manually to determine the time required for bleb formation and retraction for both dynamic and apoptotic blebs. Bleb size was followed from the time a bleb was produced until it fully retracted. Formation time was then calculated from the beginning frame to the frame when bleb size was the largest. The retraction time was calculated from the frame of the largest bleb size to the frame when the bleb was fully retracted back to the cell body.

### 2.6. Cytoskeleton distribution during dynamic and apoptotic blebbing

Dynamically blebbing cells were studied immediately after plating on Matrigel coated dishes, while apoptotically blebbing cells were studied after 1.5 h of incubation on non-coated dishes, which do not enable attachment. After incubation, dynamic and apoptotic blebbing cells were collected and centrifuged at 1100 rpm for 3 min. Both types of cells were resuspended and fixed in 4% paraformaldehyde/PBS for 15 min. After washing the pellet with PBS, cells were incubated with blocking solution containing 10% normal serum (from the same species as secondary antibody) in PBS and 0.1% Triton X-100 for 30 min at room temperature. Cells were washed with PBS and incubated with 1% BSA/PBS for 20 min at room temperature. Cells were incubated with phalloidin-Alexa 488 (Life Technologies, Grand Island, NY) or anti-tubulin-Alexa 555 (Cell Signaling Technology, Danvers, MA) for 20 min at room temperature. After washing all groups with PBS, nuclei were stained with 4′ 6-diamidino-2-phenylindole (DAPI), and cells were imaged with a Nikon Eclipse T1 microscope equipped with Elements software. For unconjugated primary antibodies, cells were incubated with rabbit anti-ezrin antibody (Epitomics, Burlingame, CA) or rabbit anti-non-muscle myosin antibody (Sigma-Aldrich, St Louis, MO) at 4 °C overnight, then washed two times the next day, and incubated with secondary antibodies conjugated with Alexa Fluorophore (goat anti-rabbit IgG secondary antibody, Alexa Fluor 594 conjugate, Sigma-Aldrich, St Louis, MO) at room temperature for 1 h. After washing with PBS, nuclei were stained with DAPI, and cells were imaged with a Nikon Eclipse T1 with Elements deconvolution software.

### 2.7. Experimental evaluation of the cytoskeleton in dynamic and apoptotic blebbing

To examine the role of the cytoskeleton in blebbing cells, single hESC, prepared as described above, were replated on Matrigel-coated 35 mm dishes containing complete mTeSR medium or drug treatments. Dishes containing dynamically blebbing single cells were immediately placed in the BioStation IM and cells were treated with either cytochalasin D (0.5 and 2 μg/ml), latrunculin A (6.25 μM), swinholide A (100 nM), nocodazole (1 μg/ml), or blebbistatin (10 μM), then incubated for live cell imaging where blebbing and attachment were followed for 4 h at 1.5 min intervals.

For experiments with dynamic and apoptotic cells, five fields were picked for each control and treatment group, and each experiment was repeated three times. To quantify the blebbing and attached cells during first 100 min of incubation in control and treated groups, cells were classified as blebbing or attached based on their morphology and dynamic behavior. The percentage of blebbing and attached cells was counted every 20 min for 100 min. 3 or 4 videos were analyzed for each treatment and control group.

### 2.8. Experimental evaluation of different matrices in hESC culture

To examine how different matrices affect dynamic blebbing and cell attachment in hESC culture, single hESC, prepared as described above with 5000 cell/35 mm dish, were replated on Matrigel, laminin-521 (BioLamina, Sweden, NR.) or Matrigel with laminin-111 (Sigma-Aldrich, St Louis, MO) coated 35 mm dishes containing complete mTeSR medium. Dishes containing suspended dynamically blebbing single cells were immediately placed in a BioStation IM for 4 h and images were collected at 3 min intervals. To quantify the blebbing and attached cells during first 60 min of incubation on different matrixes, cells were classified as blebbing or attached based on their morphology and dynamic behavior. The percentage of blebbing and attached cells was counted every 12 min for 60 min. 3 or 4 videos were analyzed for each group.

To determine if integrin and focal adhesion kinase (FAK) play roles in dynamic blebbing and attachment, hESC were treated with an α6 integrin function blocking antibody or FAK inhibitor 14. Cells were pre-incubated with the antibody or FAK inhibitor 14 for 1 h, taken off their plate with Accutase, then incubated in mTeSR medium with the α6-integrin function blocking antibody or FAK inhibitor 14 in the BioStation IM where blebbing and attachment to Matrigel were imaged for 6 h. The percentages of blebbing and attached cells were counted every 3 min for 60 min. 3 videos were analyzed in each of three independent experiments.

## 3. Statistical analysis

**Fig. 1.**
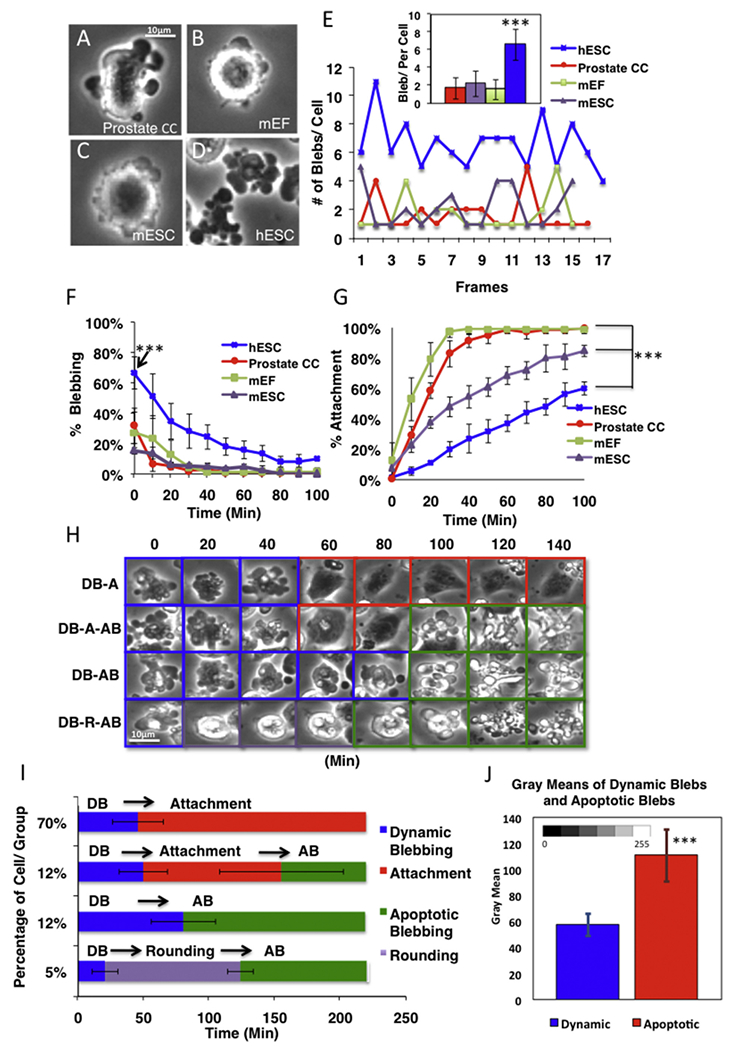
Comparison of dynamic blebbing in four cell types. All cell types produced dynamic blebs: (A) human prostate cancer cell (HU145), (B) mouse embryonic fibroblast (mEF), (C) mouse embryonic stem cell (mESC), and (D) human embryonic stem cell (hESC). (E) Number of blebs/cell in different cell types in each frame before attachment. One of three independent experiments is shown. The insert shows the number of blebs/cell averaged over all frames in all three experiments. The number of blebs/cell was significantly higher in hESC than in the other cell types by one-way ANOVA. (F, G) The percentage of blebbing cells (F) and attached cells (G) for the four cell types over 100 min. Videos were collected using a BioStation IM with 62 s intervals for hESC, 60 s intervals for prostate cancer cells and mEF, and 120 s intervals for mESC. The percentage of blebbing cells was significantly higher at the initial time point for hESC than for the other cell types (F). The percentage of attached ESC was significantly lower than the other cells at the final time point (G). F and G were analyzed by two-way ANOVA. *** = *p* < .001. (H, I) Morphological and temporal comparisons of four patterns of blebbing behavior in hESC. (H) Phase contrast images of hESC at various times in culture showing the four patterns of behavior (DB = dynamic blebbing; A = attached; AB = apoptotic blebbing; R = rounding). Dynamic blebs appear dense, while apoptotic blebs are bright. (I) Percentage of cells in each of the behavior groups shown in H over 225 min of incubation. Each group is based on a count of 25 cells. The average time to attachment, rounding, or apoptotic blebbing ± the standard deviation is shown. The percentage of cells in each group is given on the Y axis. (J) Gray means of dynamic blebs and apoptotic blebs were analyzed using ImageJ followed by a *t*-test. *** *p* < .001.

**Fig. 2.**
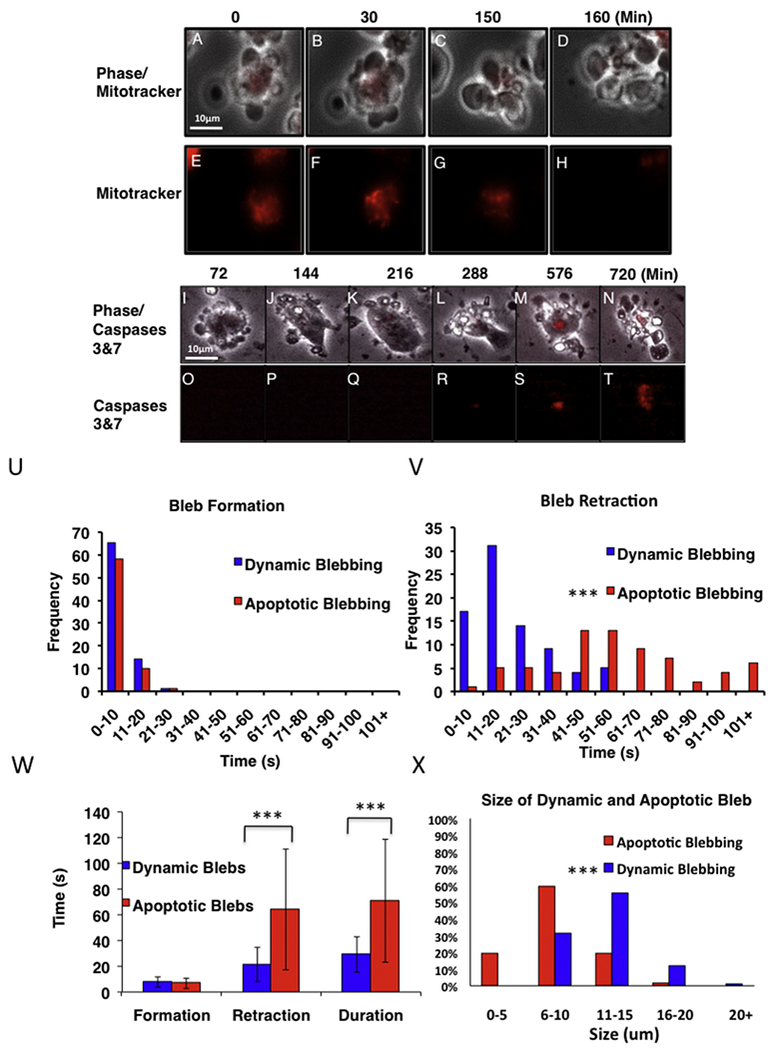
Verification of cell death and comparison of expansion and retraction times. (A-H) Phase contrast (A-D) and fluorescence images (*E*-H) at various times in culture showing that dynamically blebbing cells (E-G) have a strong fluorescence which is lost in apoptotic cells (H). (I-T) Phase contrast (I–N) and fluorescence images (O-T) at various times in culture showing cells preloaded with Magic Red. Dynamically blebbing and attached cells (I-J and O–P) do not show fluorescence. Cells undergoing apoptosis (L-N and R-T) have red fluorescence indicative of activation of caspase 3 & 7. (U) The frequency of dynamic and apoptotic blebs in various time intervals of bleb formation. (V) The frequency of dynamic and apoptotic blebs in various time intervals of retraction. Both frequency graphs are based on 80 dynamic and 69 apoptotic cells from three experiments. (W) Comparison of the time for formation, retraction and formation plus retraction (duration) for dynamic and apoptotic blebs. Each bar is the mean ± the standard deviation of three experiments. Groups were compared using a t-test. *** = p < .001. (X) Size comparison of dynamic and apoptotic blebs. Graph is based on 100 apoptotic and 100 dynamic cells from three different experiments. Frequency distributions in U, V and X were compared using Chi-square and were significantly different in V and X. *** = p < .001. (For interpretation of the references to colour in this figure legend, the reader is referred to the web version of this article.)

GraphPad Prism (GraphPad, San Diego, CA, USA) was used for all statistical analyses. Frequency distributions were compared by Chi X^2^ (Fig 2U, V, and X). One-way analysis of variance (ANOVA) was used to find significant differences in Fig. 1E insert. Statistical significance was evaluated using a two-way ANOVA in Figs. 1F, G, 5A–F, 6A–B, 6F–I and Supplemental Figs. 4 and 7. Unpaired *t*-tests were used in Figs. 1J, 2W, 6E, and Supplemental Figs. 1 and 6.

## 4. Results

### 4.1. hESC produced more dynamic blebs and blebbed longer than other cell types

Human prostate cancer cells, mEF, mESC, and hESC were dissociated from their culture dishes, transferred to new dishes, and followed for 100 min in a BioStation IM. In time-lapse videos, all four types of cells underwent blebbing before attachment to their substrates (Fig. 1A–D, Supplemental Movies 1–4). This was interpreted to be dynamic blebbing since cells blebbed, attached, and survived. In two-dimensional images, hESC produced 4–11 large blebs/cell at all times before attachment, while only 1–5 large blebs/cell were observed in the other three cell types (Fig. 1E). When the average number of blebs/cell was compared over all frames in each group, hESC had significantly more blebs/cell (6/cell) than the other three cell types (1–2/cell) (*p* < .001) (Fig. 1E insert).

The percentage of blebbing and attached cells was counted every 20 min for 100 min after plating cells on their substrates. Initially, 70% of the hESC underwent dynamic blebbing before attachment, which was significantly more than the 10–30% observed for the other cell types (p < .001) (Fig. 1F). Not only did a higher percentage of hESC bleb initially, but hESC continued to bleb longer than the other cells. When cells stopped blebbing, they attached and spread on their substrates (Fig. 1G). hESC were slower to attach than the other cell types, and by 100 min significantly fewer hESC had attached (p < .001). Similar results were seen with Riv9 iPSC (Supplemental Fig. 1 and Supplemental Movie 5).

### 4.2. Dynamic blebbing and apoptotic blebbing were morphologically and temporally distinct

Time-lapse videos of hESC were collected during the first 240 min after plating on Matrigel (Fig. 1H, I). Single hESC exhibited four different patterns of behavior following plating. Most hESC (70%) underwent dynamic blebbing (Fig. 1H, I DB-A 0–40), then attached (Fig. 1H, I DB-A 60-140), and remained attached for the duration of the 240-minute incubation. Some hESC (12%) underwent dynamic blebbing (Fig. 1H, I DB-A-AB 0–40), attached (Fig. 1H, I DB-A-AB 60-80), then detached from the substrate, and underwent apoptotic blebbing (Fig. 1H, I DB-A-AB 100-140). In the third category, cells (12%) underwent dynamic blebbing (Fig. 1H, I DB-AB 0-60) followed directly by apoptotic blebbing without attaching (Fig. 1H, I DB-AB 80-140). In the final group, cells (5%) underwent a brief period of dynamic blebbing (Fig. 1H, I DB-R-AB 0) followed by rounding (DB-R-AB 20-60), and then underwent apoptotic blebbing (DB-R-AB 80-140). In all cases, dynamic and apoptotic blebbing were well separated in time and could be distinguished morphologically. Dynamic blebbing was observed during the first hour after plating, while apoptotic blebbing, if it occurred, was observed at least 90 min after plating (Fig. 1I). When viewed with phase contrast microscopy, dynamic blebs were generally dark, while apoptotic blebs were bright. Bleb brightness was quantified using Image *J*, and dynamic blebs were quantitatively significantly darker than apoptotic blebs (Fig. 1J).

To confirm the above interpretations, hESC labeled with either Mitotracker Red, which fluoresces in healthy mitochondria (Fig. 2A–H), or with Magic Red, which fluoresces when caspases 3&7 are activated (Fig. 2I–T). During the dynamic blebbing interval (0–150 min in Fig. 1A–C), Mitotracker Red was highly fluorescent and localized in cell bodies, not in blebs, indicating cells were healthy and that mitochondria had an intact membrane potential (Fig. 2A–C and E–G). However, by 2.5 h, the same cells showed diminished fluorescence, indicating damage to the mitochondria and probable loss of mitochondrial membrane potential (Fig. 2D, H), which is characteristic of apoptotic cells (Gottlieb et al., 2003). Dynamically blebbing cells did not fluoresce when incubated with Magic Red (Fig 2I–K and O–Q); however, after these cells had incubated 288 min in dishes that did not permit attachment, activated caspases 3&7 were detected, and subsequently apoptotic blebs appeared (Fig. 2L-N and R-T). The data with Mitotracker Red and Magic Red confirmed that dynamically blebbing cells were not undergoing apoptosis and that cells blebbing late in the incubation interval were indeed apoptotic.

### 4.3. Rate of retraction and size differ in dynamic and apoptotic blebs

The rates of formation and retraction of dynamic and apoptotic blebs were analyzed using real time videos of cells that were freshly plated on Matrigel (dynamic blebbing) and cells that had incubated on uncoated dishes for 1.5 h (apoptotic blebbing) (Fig. 2U–W and Supplemental Movies 6 and 7). The average time for dynamic (8 s for 80 cells) and apoptotic (7 s for 69 cells) bleb formation was not statistically different by Chi^2^ analysis (Fig. 2U). However, apoptotic blebs took significantly longer (*p* < .001) to retract (average = 64 s for 69 cells) than dynamic blebs (average = 22 s for 80 cells) (Fig. 2V), and therefore the overall duration of dynamic and apoptotic blebbing was significantly different (*p* < .0001) (Fig. 2W). In some apoptotic cells, retraction had not occurred by 6 min (not shown). >100 randomly chosen blebs were analyzed for area (microns) using ImageJ. Dynamic and apoptotic bleb size differed significantly (p < .001) with dynamic blebs being generally larger than apoptotic blebs (Fig. 2X).

### 4.4. Distribution of the cytoskeleton during dynamic and apoptotic blebbing

**Fig. 3.**
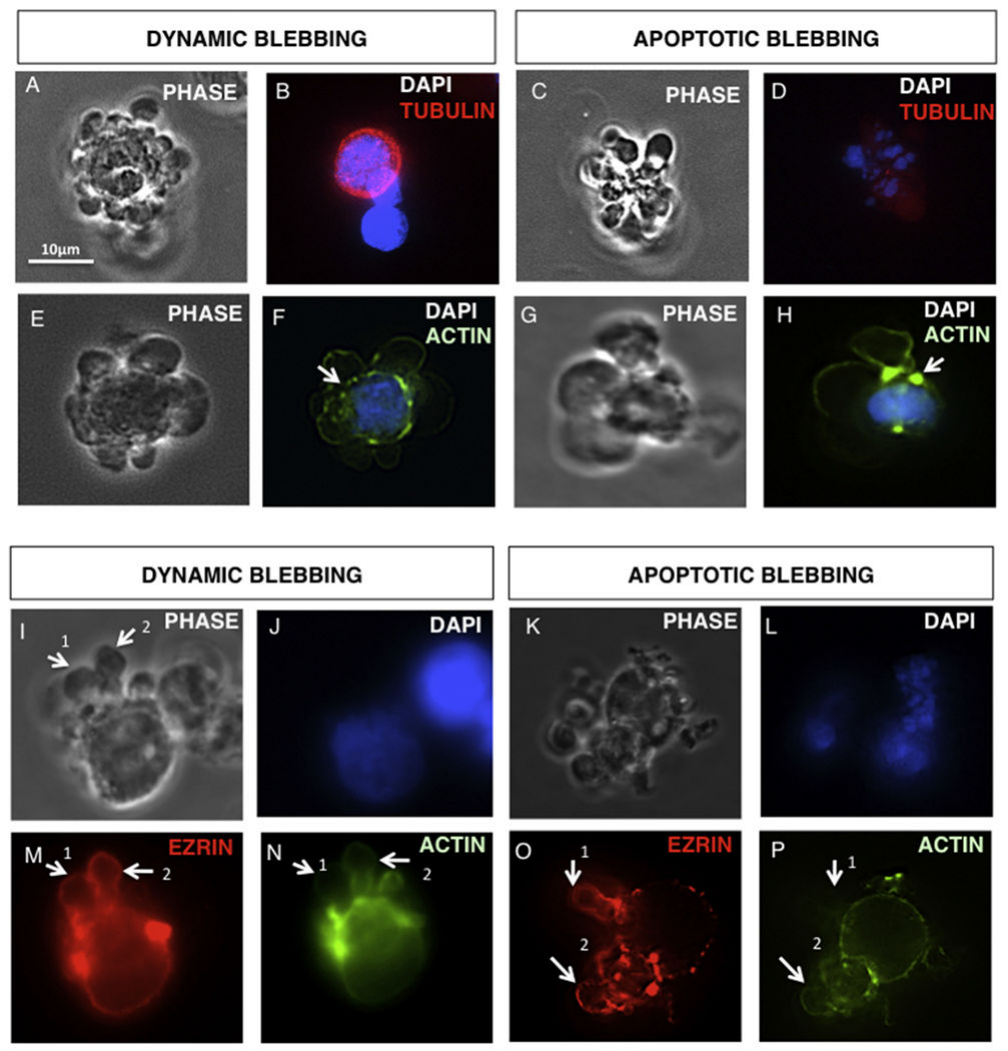
Distribution of the tubulin, actin and ezrin during bleb formation in dynamic and apoptotically blebbing cells. (A-D) Phase and fluorescent micrographs showing the distribution of microtubules in dynamic (A, B) and apoptotic blebs (C, D). (E-H) Phase and fluorescence micrographs showing the distribution of actin filaments in dynamic (E, F) or apoptotic blebs (G, H) during bleb formation. Arrows indicate breaks in the cortical actin ring of dynamically blebbing cells (F) or hot spots of depolymerized actin filaments in apoptotically blebbing cells (H). (I–P) Phase and fluorescent micrographs showing the localization of ezrin and actin in expanding dynamic blebs (I, J, M, N) and apoptotic blebs (K, L, O, P).

During formation of dynamic blebs, microtubules formed a continuous thick uninterrupted band around the cell periphery and did not extend into the blebs (Fig. 3A, B). In contrast, apoptotically blebbing cells with fragmented nuclei contained depolymerized tubulin (Fig. 3C, D). In dynamically blebbing cells, a cortical actin ring was present but interrupted by ruptures (arrowheads) beneath the blebs (Fig. 3E, F). In contrast, most actin in the apoptotically blebbing cells was concentrated in several hot spots (arrowheads), and a cortical band of actin was not present (Fig. 3G, H).

The relationship between actin and ezrin, an integral membrane protein that attaches actin filaments to the plasma membrane (Solinet et al., 2013), was evaluated during bleb formation (Fig. 3I and M, K and O). Ezrin was associated with the plasma membrane in non-blebbing cells (data not shown) and was localized in the plasma membrane of both dynamic and apoptotic blebs (Fig. 3M, O). Expanding dynamic bleb membranes either lacked associated actin (Fig. 3N arrowhead 1) or had a continuous band of actin subjacent to the bleb membrane (Fig. 3N arrowhead 2). A similar distribution of actin was seen in apoptotic blebs. Some apoptotic cells lacked actin beneath the bleb membranes (Fig. 3P arrowhead 1), while others had actin associated with blebs (Fig. 3 P arrowhead 2). These data suggest that actin moves into the blebs and reassembles into filaments after expansion.

**Fig. 4.**
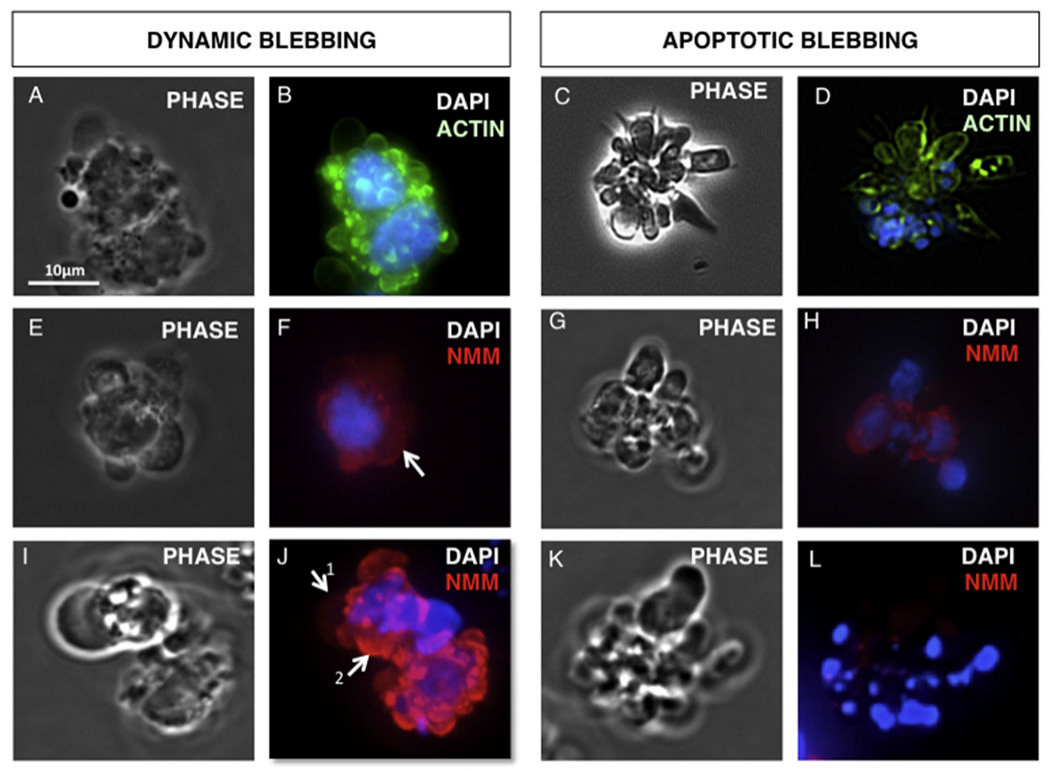
Distribution of the actin and non-muscle myosin in dynamic and apoptotically blebbing cells. (A, B) Phase and fluorescent micrographs showing the distribution of actin in retracting dynamic blebs. (C, D) Phase and fluorescent micrographs showing the distribution of actin in late apoptotic blebs. (E, F, I, J) Phase and fluorescent micrographs showing the distribution of non-muscle myosin II in non-retracting dynamic blebs (E, F) or retracting blebs (I, J). (G, H, K, L) Phase and fluorescent micrographs showing the distribution of non-muscle myosin II in early (G, H) and late apoptotic blebs (K, L).

During retraction, actin and myosin localization differed in dynamic and apoptotically blebbing cells (Fig. 4A–L). In dynamically blebbing cells, small retracting blebs had intense actin labeling adjacent to the bleb membrane (Fig. 4A, B). In apoptotically blebbing cells, actin was sometimes observed adjacent to bleb membranes, but was often fragmented and formed hot spots (Fig. 4C, D). In dynamic blebs, myosin was diffuse in forming blebs (Fig. 4E, F, I, J), but was reassembled with actin under membranes in small retracting blebs (Fig. 4B, I, J). In late stages of apoptosis when nuclei were highly fragmented, cells had little myosin staining (Fig. 4 G, H, K, L).

The preceding data demonstrate that dynamic and apoptotic blebbing are two distinct processes that occur during plating of hESC. These types of blebbing were further investigated using drugs that affect the cytoskeleton.

### 4.5. Nocodazole prolonged dynamic blebbing and inhibited attachment

**Fig. 5.**
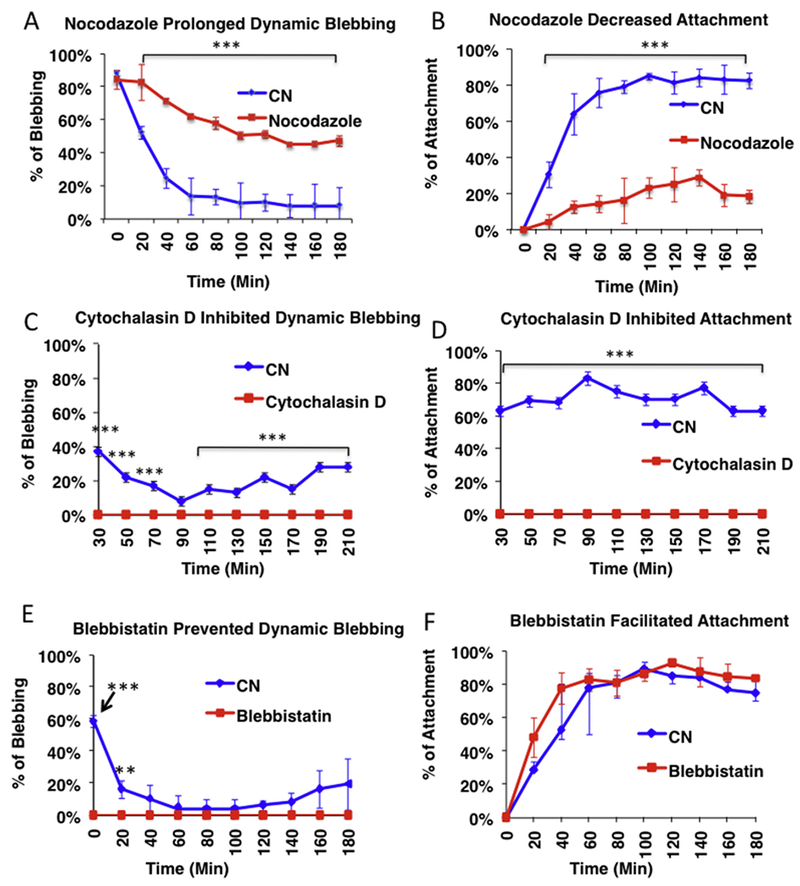
The effect of cytoskeletal drugs on dynamically blebbing cells: (A, B) 1 μg/ml nocodazole prolonged dynamic blebbing (A) and inhibited attachment (B) in hESC. (C, D) 2 μg/ml Cytochalasin D inhibited both dynamic blebbing (C) and attachment (D) in hESC. (E, F) 10 μM blebbistatin inhibited dynamic blebbing (E) and slightly accelerated attachment (F) in hESC. Each point is the mean of three experiments ± the standard deviation of three experiments. In each experiment, three videos were analyzed. All data were analyzed by a 2-way ANOVA. ** *p* < .01; *** p < .001.

To determine if microtubules played a role in dynamic blebbing and attachment, cells were treated with 1 μg/ml of nocodazole, which depolymerized microtubules (Supplemental Fig. 2A–D). BioStation time-lapse video data showed that blebbing control cells began attaching by 20 min of incubation, while nocodazole treated cells were still dynamically blebbing by 60 min (Fig. 5A, B). In quantified video data, there were significantly more (*p* < .001) dynamically blebbing cells in the nocodazole treated group than in the control at 20 min of incubation (Fig. 5A). In addition, blebbing was prolonged by nocodazole, and by 180 min most treated cells were still dynamically blebbing (p < .001 for control *vs.* treated at 180 min) (Fig. 5A). Dynamically blebbing cells failed to attach to the Matrigel substrate (Fig. 5 B).

### 4.6. Cytochalasin D and blebbistatin inhibited dynamic blebbing in hESC

To determine if fragmentation of actin filaments affected dynamic blebbing, cells were treated with cytochalasin D (2 μg/ml) (Fig. 5C), latrunculin A (6.25 μM) or swinholide A (100 nM) (Supplemental Fig. 2E-L), then incubated in a BioStation IM where blebbing and attachment to Matrigel were imaged for 6h. Cytochalasin D (2 μg/ml), latrunculin A, and swinholide A depolymerized actin filament, resulting in numerous actin hot spots (Supplemental Fig. 2EμL). Dynamic blebbing was completely inhibited by cytochalasin D; cells remained round with few or no blebs and did not attach (Fig. 5D). Quantification of the video data showed that cytochalasin D inhibited both dynamic bleb formation and cell attachment (Fig. 5C-D). Inhibition of bleb formation by cytochalasin D was dose dependent (Supplemental Fig. 3 A-G). 0.5 μg/ml of cytochalasin D enabled some cells to form blebs, which did not retract, while 2 μg/ml completely inhibited bleb formation (Supplemental Fig. 3 G). Attachment was also completely inhibited by the higher dose, and partially inhibited by 0.5 μg/ml of cytochalasin D.

To determine if myosin was likewise involved in dynamic blebbing, cells were treated with blebbistatin, a myosin II inhibitor (Fig. 5E, F). None of the cells underwent dynamic blebbing after treatment with blebbistatin, and cells attached to the substrate soon after plating (Fig. 5E, F).

### 4.7. Laminin-521 inhibited dynamic blebbing, accelerated cell attachment, and reduced apoptosis

**Fig. 6.**
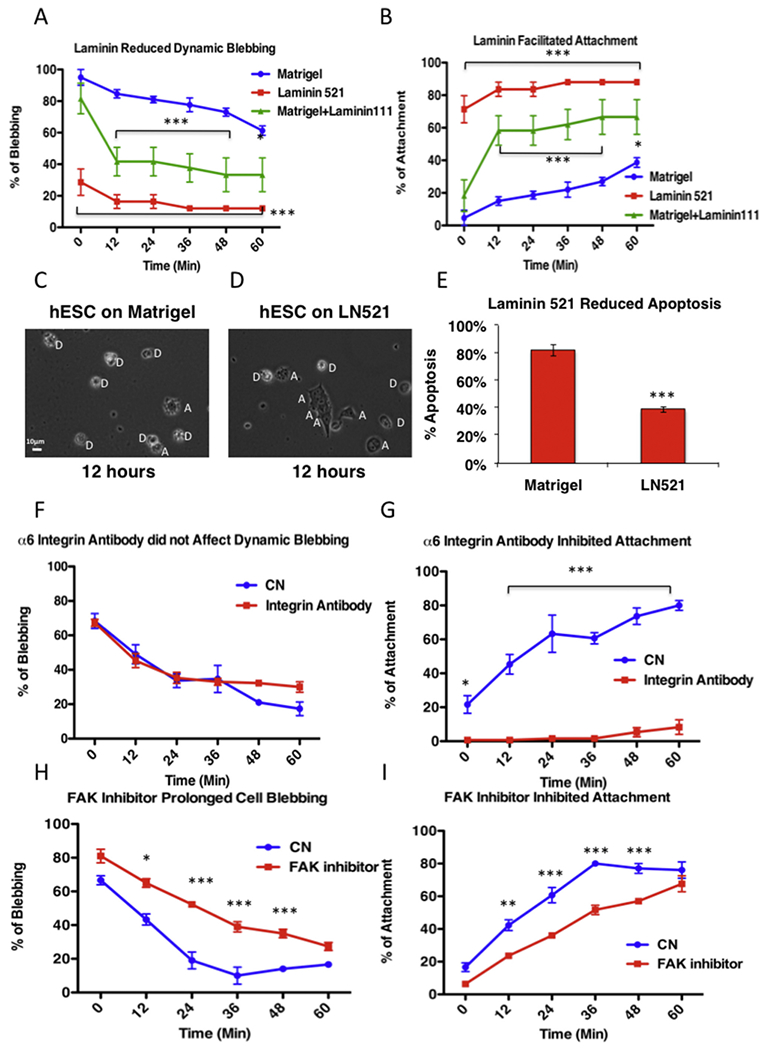
Laminin, integrin, and FAK signaling reduces dynamic blebbing and increases cell attachment/survival. (A, B) Laminin-521 and Matrigel with laminin-111 inhibited dynamic blebbing (A) and accelerated attachment (B) in hESC. (C, D) Phase images of hESC on Matrigel (C) and laminin-521 (D). White “D”s show apoptotic blebbing/dead cells; white “A”s show attached cells. (E) Quantitative data from the videos showed the percentage of apoptotic cells on Matrigel and laminin-521. Each bar is the mean of three experiments ± the standard deviation. (F, G) α6-Integrin antibody did not affect dynamic blebbing (F) but inhibited attachment (G) in hESC. (H, I) FAK inhibitor prolonged dynamic blebbing (H) and inhibited attachment (I) in hESC. Graphical data were analyzed by 2-way ANOVA. Each point is the mean ± standard deviation of three experiments. In each experiment, three videos were analyzed. * *p* < .05; ** p < .01; *** p < .001.

To improve plating efficiency and hESC survival, we sought a safe non-invasive method that did not rely on xenobiotics or inhibitors to diminish or reverse dynamic blebbing and promote cell attachment. To do this, we evaluated dynamic blebbing and attachment on different matrices that are used for hESC culture. Dynamic blebbing was significantly inhibited and cell attachment was accelerated when cells were plated on recombinant laminin-521 (Fig. 6A, B). Similar results were obtained when RIV9 iPSC were incubated on laminin-521 (Supplemental Fig. 4; Supplemental Movie 8). Because Matrigel contains laminin-111 (Gottlieb et al., 2003), we also tested cells plated on Matrigel over-coated with additional laminin-111. The combination of laminin-111 and Matrigel also significantly reduced dynamic blebbing and accelerated attachment, although this combination was not as efficient as laminin-521 alone (Fig. 6A, B). Individual cells were monitored in the videos, and apoptotic cells were identified and counted based on their morphology (Fig. 6C, D). Apoptotically dying cells have bright blebs with slow retraction times. Cells plated on laminin-521 had significantly fewer apoptotic cells than the group plated on Matrigel (Fig. 6E). In addition, cells plated on Matrigel had activated caspase 3&7, while those on laminin-521 did not (Supplemental Fig. 5).

We next investigated the hypothesis that binding of hESC to laminin activates signaling through an integrin and FAK, which shuts down dynamic blebbing. Cells were pre-incubated with an α6-integrin function blocking antibody for 1 h, taken off the plate with Accutase, then incubated in medium containing the α6 integrin-function blocking antibody and imaged in a BioStation IM where blebbing and attachment to Matrigel were followed for 6 h. Dynamic blebbing was not significantly affected by the integrin α6-function blocking antibody (Fig. 6F). However, cell attachment was significantly inhibited (Fig. 6G) and 56% of cells were rounded after 1 h in the α6-integrin-function blocking antibody (Supplemental Fig. 6).

To determine if integrin signaled through a FAK, cells were pre-incubated with a FAK inhibitor for 1 h, taken off the plate with Accutase, then incubated with the inhibitor and imaged in the BioStation IM, where blebbing and attachment to Matrigel or laminin-521, were imaged for 6 h. Quantification of the video data showed that FAK inhibitor prolonged dynamic blebbing and slowed cell attachment on Matrigel and laminin-521 (Fig. 6H, I and Supplemental Fig 7 A, B).

## 5. Discussion

A distinction between dynamic and apoptotic blebbing has not been made previously for freshly plated hESC. Dynamic and apoptotic blebbing were well separated in time, and also differed in their rates of retraction, bleb intensity, assembly of cytoskeletal proteins, distribution of organelles during blebbing, mitochondrial membrane potential (MitoTracker), activation of caspases 3&7, and their response to chemicals that affect the cytoskeleton. During passaging onto Matrigel, hESC underwent prolonged dynamic blebbing that inhibited attachment and spreading and stressed the cells. Typically, dynamic blebbing subsided within an hour of plating allowing cell attachment and spreading to occur. hESC that failed to attach, eventually underwent apoptotic blebbing and died. These data demonstrate that dynamic blebbing is a critical bottleneck that decreases efficient passaging of hESC and precludes passaging of single cells. Gaining control of dynamic blebbing is necessary in hESC research and in the application of hESC therapies to patients.

**Fig. 7.**
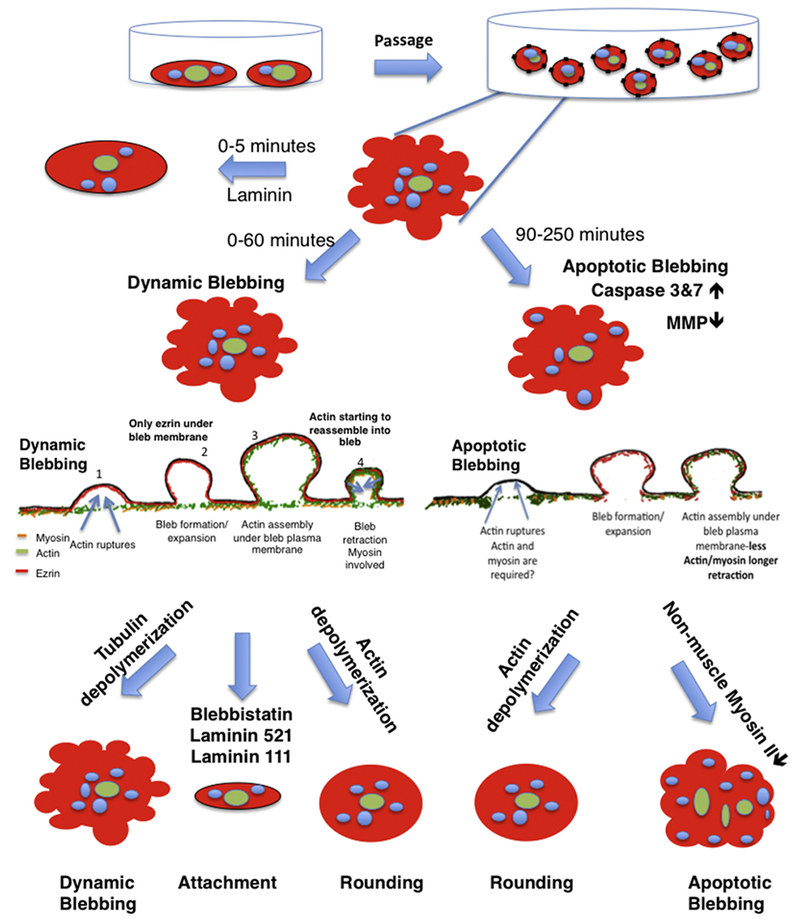
Diagram comparing dynamic and apoptotic blebbing in hESC and summarizing the main findings of the study.

Fig. 7 summarizes our data comparing dynamic and apoptotic blebbing in freshly plated hESC. When bundles of cortical actin ruptured, the plasma membrane detached from the actin and blebs formed. During bleb expansion, ezrin remained associated with the bleb membrane, and actin was not attached to ezrin at this time. As the blebs expanded, actin moved into the blebs, attached to ezrin, and re-assembled under the bleb membrane. In dynamic blebbing, myosin reassembled with actin in the bleb, and their contraction causes bleb retraction. In apoptotic blebs, actin and myosin were fragmented, less abundant, or absent, which may account for their slower retraction. Depolymerizing microtubules prolonged dynamic blebbing and inhibited attachment. Use of blebbistatin or plating on laminin-521 or 111 decreased dynamic blebbing and facilitated cell attachment.

The preceding observations raise two questions about dynamic blebbing. What is the significance of dynamic blebbing and why does it occur so vigorously in hESC? Cells dissociated from amphibian embryos can migrate using bleb-like protrusions (Holtfreter, 1943; Kubota, 1981; Satoh et al., 1976), and live zebrafish have primordial germ cells that use blebs to migrate (Blaser et al., 2006; Trinkaus, 1972; Wourms, 1972; Trinkaus, 1996). Similar observations have been made in primordial germ cells from *Drosophila melanogaster* embryos (Jaglarz and Howard, 1995). These findings suggest that dynamic blebbing is widely used for cell migration and further suggest that dynamic blebbing may be a common mechanism for generating motility in embryonic cells.

Many cells undergo dynamic blebbing during plating, but hESC are unusual in producing more blebs for a longer time than the other cell types we tested. hESC therefore appear programmed to undergo hyperdynamic blebbing. When cultured *in vitro*, hESC behave like epiblast cells, a highly motile epithelialized type of embryonic cell (Ohgushi and Sasai, 2011; Nichols and Smith, 2012). During weeks 2 and 3 of human development, sheets of epiblast cells undergo extensive migration, and during gastrulation, they enter the primitive streak, pinch off of the groove, and migrate as single cells to form both the endoderm and mesoderm (Moore, 2016). It has been suggested that hESC blebbing is driven by hyperactivity of the ROCK/myosin system (Ohgushi et al., 2010; Ohgushi and Sasai, 2011). It is possible that in embryos dynamic blebbing provides motility to epiblast cells during gastrulation. In contrast, mESC, which resemble the inner cell mass, did not undergo extensive blebbing (Fig. 1A), indicating that earlier stages in development have a less active cytoskeleton.

Dynamic blebbing has important consequences for cultured hESC. First, cells that are dynamically blebbing cannot attach to their matrix until blebbing stops. Secondly, cells that are dynamically blebbing appear severely stressed and often shed part of their cytoplasm in the form of blebs that pinch off rather than retract. Therefore, factors that reduce dynamic blebbing and accelerate attachment would benefit hESC culture. ROCKi (Y27632) is often used to help hESC attach and spread rapidly; however, ROCKi treated cells often appear stringy and stressed (Weng et al., 2015). Furthermore, 20 μM ROCKi can promote the differentiation of mESC into motor and sensory neurons (Kamishibahara et al., 2014). ROCKi may produce other unwanted off-target effects that are not desirable when cells are being prepared for research, translational, or clinical use. Blebbistatin is also used to accelerate attachment by inhibiting myosin II, but again it is not a normal additive to culture media, and it may affect processes in addition to blebbing, such as cytokinesis (Guha et al., 2005).

For hESC culture, plating on laminin-521 or Matrigel over- coated with laminin-111 improves cell attachment and survival by reducing dynamic blebbing and enables efficient plating of single cells, while concurrently incubating in conditions that resemble the cells’ natural *in vivo* environment without inhibitors that may have unwanted off-target effects. Cells maintain pluripotency and do not express differentiation markers during repeated plating on laminin-521 (Supplemental Fig. 8 A-J). We showed that plating hESC on recombinant human laminin-521, a biological molecule present in the inner cell mass (Schilperoort-Haun and Menino Jr., 2002), signals through an integrin-FAK pathway to stop dynamic blebbing and accelerate attachment, thereby enabling cell survival. Without attachment, hESC undergo apoptotic blebbing and survive a relatively short time and in culture.

The integrin-FAK pathway plays a number of important roles in hESC where its activation functions in apparently non-canonical ways to affect cell survival, self-renewal, and the maintenance of pluripotency (Vitillo and Kimber, 2017). Our data are in agreement with another study showing that FAK signaling promotes stem cell survival by inhibiting hyper-contractility (dynamic blebbing) (Vitillo et al., 2016). Following successful attachment, FAK activation further promotes survival though a pathway leading to degradation of P53 (Vitillo and Kimber, 2017). Inhibition of dynamic blebbing by FAK activation is crucial to successful plating and passaging of hESC and can be used to advantage to improve hESC culture protocols important for both basic and translational research.

## 6. Conclusion

Dynamic blebbing interferes with attachment and survival of hESC during passaging. Cells that fail to attach eventually undergo apoptotic blebbing and die. Plating efficiently can be improved by passaging onto laminin which activates a FAK that in turn stops blebbing, facilitates attachment and spreading, and allows repeated passaging of single cells. This strategy provides a safe means for passaging human pluripotent stem cells that does not require drugs with off target effects. The role of FAK in human pluripotent stem cells deserves further work and may lead to major improvements in optimization of pluripotent stem cell culture.

## Supplementary Material

Supplemental Figure 1

Supplemental Movie 2

Supplemental Movie 3

Supplemental Movie 4

Supplemental Movie 5

Supplemental Movie 6

Supplemental Movie 7

Supplemental Movie 8

Supplemental Figure 2

Supplemental Figure 3

Supplemental Figure 4

Supplemental Figure 5

Supplemental Figure 6

Supplemental Figure 7

Supplemental Figure 8

Supplemental Movie 1
