# Supplementary figures and images for: Dynamic blebbing: A bottleneck to human embryonic stem cell culture that can be overcome by Laminin-Integrin signaling
Source: Stem Cell Res. Author manuscript; Available in PMC 2019 Mar 12. (PMC6414319; doi:10.1016/j.scr.2018.10.022)

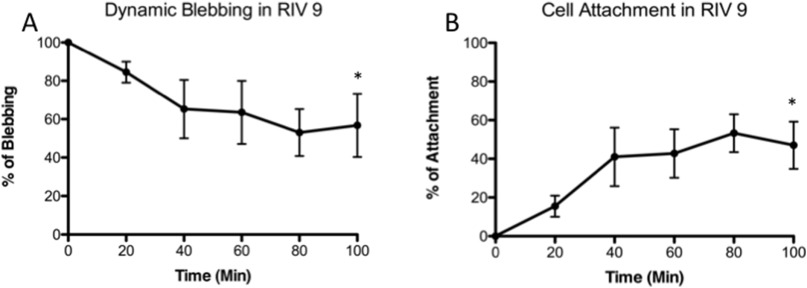

Supplement: Supplemental Figure 1 [file NIHMS1516446-supplement-Supplemental_Figure_1.jpg]

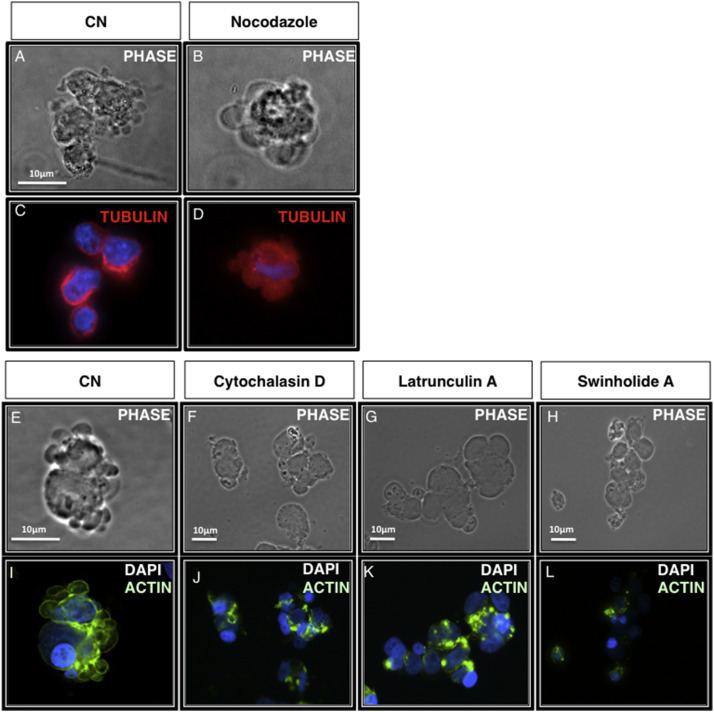

Supplement: Supplemental Figure 2 [file NIHMS1516446-supplement-Supplemental_Figure_2.jpg]

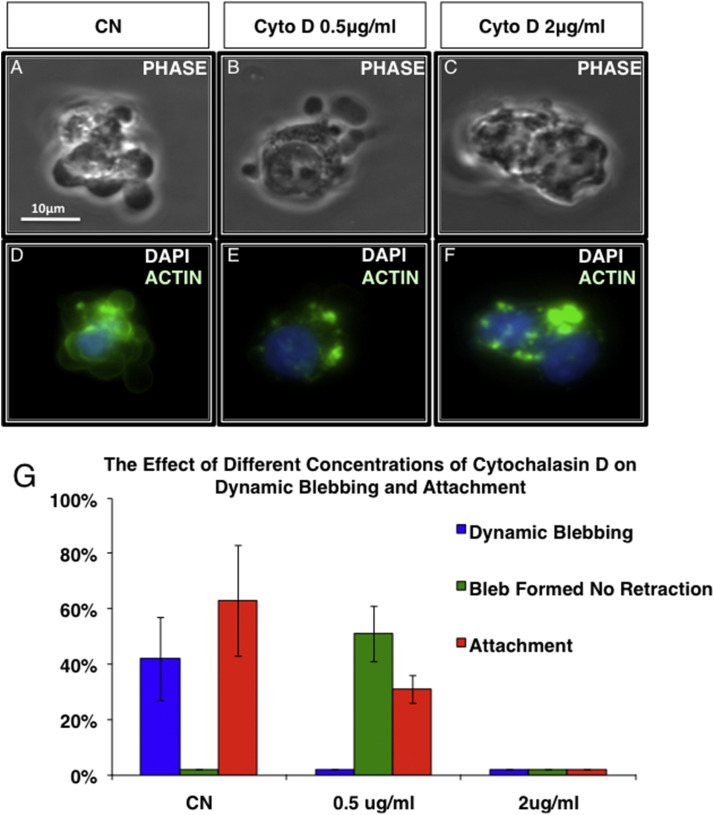

Supplement: Supplemental Figure 3 [file NIHMS1516446-supplement-Supplemental_Figure_3.jpg]

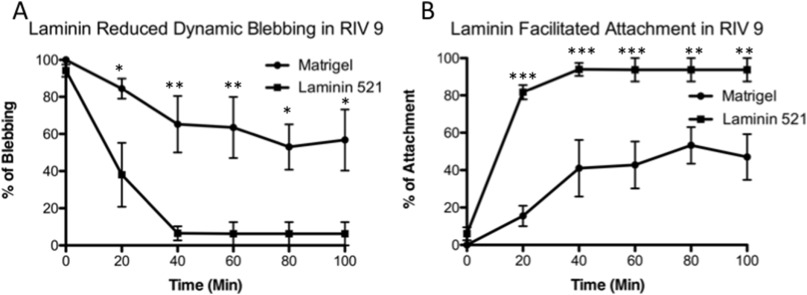

Supplement: Supplemental Figure 4 [file NIHMS1516446-supplement-Supplemental_Figure_4.jpg]

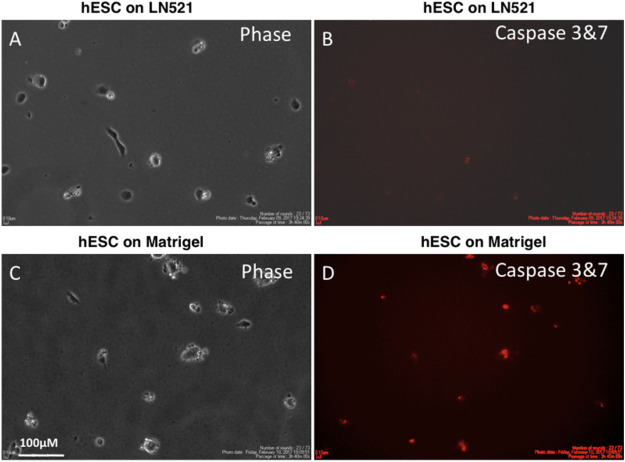

Supplement: Supplemental Figure 5 [file NIHMS1516446-supplement-Supplemental_Figure_5.jpg]

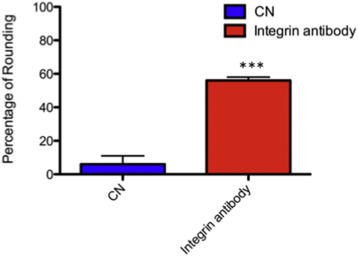

Supplement: Supplemental Figure 6 [file NIHMS1516446-supplement-Supplemental_Figure_6.jpg]

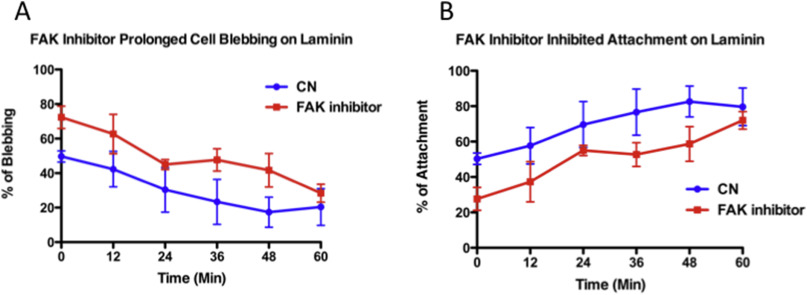

Supplement: Supplemental Figure 7 [file NIHMS1516446-supplement-Supplemental_Figure_7.jpg]

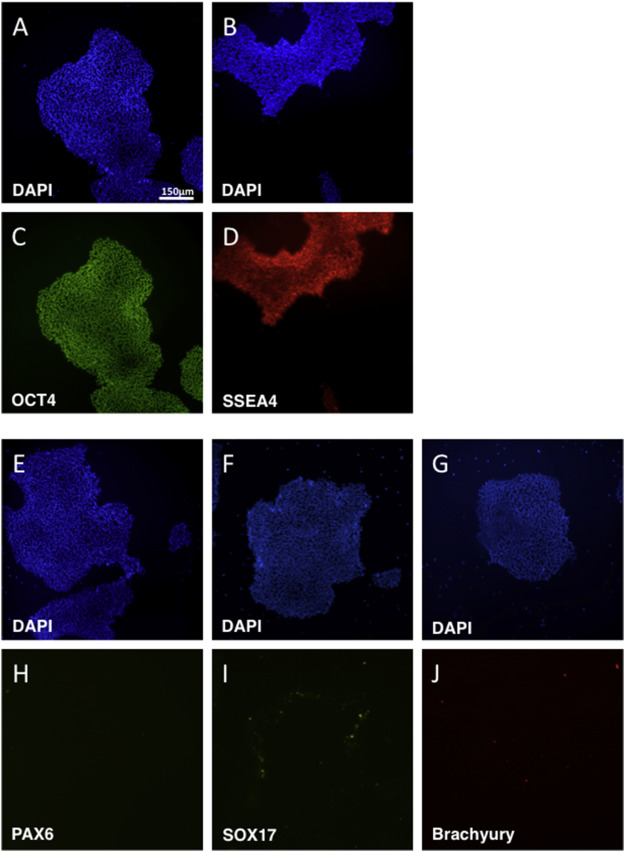

Supplement: Supplemental Figure 8 [file NIHMS1516446-supplement-Supplemental_Figure_8.jpg]
